# Effect of liraglutide on cardiac function in patients with type 2 diabetes mellitus: randomized placebo-controlled trial

**DOI:** 10.1186/s12933-019-0857-6

**Published:** 2019-04-30

**Authors:** Maurice B. Bizino, Ingrid M. Jazet, Jos J. M. Westenberg, Huub J. van Eyk, Elisabeth H. M. Paiman, Jan W. A. Smit, Hildebrandus J. Lamb

**Affiliations:** 1 Department of Radiology, Leiden University Medical Center, LUMC Postzone C2S, Albinusdreef 2, 2333 ZA Leiden, The Netherlands; 2 Department of Medicine, Division of Endocrinology, Leiden University Medical Center, Leiden, The Netherlands; 3 Department of Medicine, Radboud University Medical Center, Nijmegen, The Netherlands

**Keywords:** Diabetes mellitus type 2, Liraglutide, GLP1-receptor agonist, Diastolic heart failure, Cardiac function

## Abstract

**Background:**

Liraglutide is an antidiabetic agent with cardioprotective effect. The purpose of this study is to test efficacy of liraglutide to improve diabetic cardiomyopathy in patients with diabetes mellitus type 2 (DM2) without cardiovascular disease.

**Methods:**

Patients with DM2 were randomly assigned to receive liraglutide 1.8 mg/day or placebo in this double-blind trial of 26 weeks. Primary outcome measures were LV diastolic function (early (E) and late (A) transmitral peak flow rate, E/A ratio, early deceleration peak (Edec), early peak mitral annular septal tissue velocity (Ea) and estimated LV filling pressure (E/Ea), and systolic function (stroke volume, ejection fraction, cardiac output, cardiac index and peak ejection rate) assessed with CMR. Intention-to-treat analysis of between-group differences was performed using ANCOVA. Mean estimated treatment differences (95% confidence intervals) are reported.

**Results:**

23 patients were randomized to liraglutide and 26 to placebo. As compared with placebo, liraglutide significantly reduced E (− 56 mL/s (− 91 to − 21)), E/A ratio (− 0.17 (− 0.27 to − 0.06)), Edec (− 0.9 mL/s^2^ * 10^−3^ (− 1.3 to − 0.2)) and E/Ea (− 1.8 (− 3.0 to − 0.6)), without affecting A (3 mL/s (− 35 to 41)) and Ea (0.4 cm/s (− 0.9 to 1.4)). Liraglutide reduced stroke volume (− 9 mL (− 16 to − 2)) and ejection fraction (− 3% (− 6 to − 0.1)), but did not change cardiac output (− 0.4 L/min (− 0.9 to 0.2)), cardiac index (− 0.1 L/min/m^2^ (− 0.4 to 0.1)) and peak ejection rate (− 46 mL/s (− 95 to 3)).

**Conclusions:**

Liraglutide reduced early LV diastolic filling and LV filling pressure, thereby unloading the left ventricle. LV systolic function reduced and remained within normal range. Future studies are needed to investigate if liraglutide-induced left ventricular unloading slows progression of diabetic cardiomyopathy into symptomatic stages.

*Trial registration* ClinicalTrials.gov: NCT01761318.

## Introduction

Patients with type 2 diabetes mellitus (DM2) are at increased risk for heart failure, even in the absence of coronary artery disease and hypertension. This so-called diabetic cardiomyopathy is characterized by left ventricular (LV) diastolic dysfunction [1] and has an estimated prevalence of approximately 50% [2]. When heart failure symptoms have developed, most patients with diabetic cardiomyopathy are classified as heart failure with preserved ejection fraction (HFpEF). HFpEF poses patients with DM2 at a very high morbidity [3] and mortality risk [4]. Therefore, early detection followed by medical therapy to reverse LV diastolic dysfunction seems an attractive goal in diabetes management. Although intense glycaemic control is a primary tool to reduce diabetes complications, tight glucoregulation by itself does not seem to improve LV diastolic function [5]. Nor are there any specific drugs besides diuretics that can be used to treat or prevent HFpEF [6].

The anti-diabetic agent liraglutide is a glucagon-like peptide-1 receptor agonist (GLP-1RA) that improves insulin secretion, suppresses glucagon production and induces weight loss. Although some studies have investigated the effect of GLP-1RA on ischemic heart disease and symptomatic heart failure with reduced ejection fraction (HFrEF) [7], little is known about the effect on LV diastolic function. GLP-1RA induced weight loss by itself might improve LV diastolic function [8]. In addition, a direct cardio-protective effect of GLP-1RA therapy has been suggested by preclinical studies and in some but not all human studies [9].

Cardiac magnetic resonance imaging (CMR) has been shown to enable accurate assessment of LV diastolic and systolic function with very high reproducibility [10, 11]. Therefore, the purpose of this randomized placebo controlled trial was to evaluate the effect of the GLP1-RA liraglutide on CMR-derived indices of cardiac function in patients with DM2 without prior cardiovascular disease.

## Methods

### Study design and participants

The MAGNA VICTORIA (MAGNetic resonance Assessment of VICTOza efficacy in the Regression of cardiovascular dysfunction In type 2 diAbetes mellitus) study was an investigator-initiated randomized, double-blind, assessor-blinded, placebo-controlled, single-center clinical trial with 26 weeks follow-up. Men and women with DM2 were eligible if aged 18–69 years. Inclusion criteria were: BMI 25 kg/m^2^ or above; glycated haemoglobin (HbA1c) level of 7.0 to 10.0% (53–86 mmol/mol) despite use of maximally tolerable metformin treatment, with or without sulphonyurea derivative (SUD) and/or insulin, with stable dosage in the 3 months before study entry; blood pressure < 150/85 mmHg and stable for at least 1 month. Due to lack of eligible patients use of SUD and insulin was added to inclusion criteria after commencement of the trial. Exclusion criteria were: use of other glucose-lowering therapy than mentioned above; history or presence of renal, hepatic or cardiovascular disease; gastric bypass surgery; chronic pancreatitis or previous acute pancreatitis; pregnant or lactating women; and contra-indications for MRI. The trial was approved by the local ethics committee and performed in accordance with the principles of the revised Declaration of Helsinki. Written informed consent was obtained from all participants before study entry. The trial was conducted at the Leiden University Medical Center (LUMC), Leiden, the Netherlands, and was registered at clinicaltrials.gov (NCT01761318).

## Study procedures

### Screening visit

Participants underwent pre-screening by telephone to assess eligibility on the basis of drug use, medical history, and anthropometric measures. If potentially eligible, participants were submitted to a screening visit with detailed history taking with special interest to cardiovascular symptoms and presence of neuropathy (peripheral sensory neuropathy as detected by monofilament testing and/or erectile dysfunction), nephropathy (micro-albuminuria) and retinopathy. Height, weight and blood pressure were measured and physical examination, resting electrocardiogram (ECG) and blood examination were performed. The nonattendance of cardiovascular disease was defined as absence of symptoms related to coronary artery disease and heart failure and normal ECG.

### Randomization and masking

Patients were randomized to liraglutide (Victoza, Novo Nordisk A/S, Bagsvaerd, Denmark) or placebo (provided by Novo Nordisk A/S, Bagsvaerd, Denmark) once daily subcutaneous injections, added to their pharmacologic treatment at study entry. Participants were randomized with 1:1 stratification for sex and insulin use (block size of 4) to increase likelihood of comparable groups given the relatively low sample size. Randomization was performed by the local research pharmacist (Department of Clinical Pharmacy, LUMC, Leiden, The Netherlands) after investigator had provided information on sex and insulin use (directly after results of the screening visit). All investigators, study personnel and participants were blinded to treatment allocation until the study had been completed (including CMR post-processing and analyses).

### Study protocol

Study drug was uptitrated from 0.6 mg in the first week, 1.2 mg in the second week and 1.8 mg from week three on (if well-tolerated). Study drug dosage was reduced if necessitated by adverse events. Patients were instructed to return their used study drug pens in order to calculate compliance. In order to prevent hypoglycaemia, an individualised adjustment was made regarding concomitant glucose-lowering drugs at study entry, based on hypoglycaemic events prior to the study and HbA1c value at screening visit. Patients using insulin were encouraged to perform ambulant glucose monitoring according to clinical practice guidelines, and participants not using insulin were provided with an ambulant glucose meter to perform once weekly fasting glucose and upon hypoglycaemic symptoms. Patients had contact to study investigators once weekly by telephone, and a study visit once monthly. Weight and blood pressure (average of 2–3 measurements using automatic calibrated device in supine position) were measured at each study visit. During the study, glycaemic drugs were titrated according to clinical practice guidelines by means of dose adjustment of insulin and/or SUD. Adjustment of antihypertensive and lipid-lowering drugs were made if necessary. At study entry, week 12 and at end of study, blood examinations were performed after at least six hours of fasting. HbA1c was measured with boronate affinity high-performance liquid chromatography (Primus Ultra, Siemens Healthcare Diagnostics, Breda, the Netherlands) throughout the first part of the study. The laboratory chose to change their HbA1c measurement method for logistic reasons while our study was ongoing. The method was changed into ion-exchange high-performance liquid chromatography (HPLC) (Tosoh G8, Sysmex Nederland B.V., Etten-Leur, the Netherlands). HbA1c values were corrected on the basis of the correlation coefficient that was derived from a validation experiment that used data of 196 samples that were measured on both analysers (data can be provided on request). All other blood samples were centrifuged and stored at − 80 °C until analysis. Serum creatinine, triglyceride, total cholesterol, HDL-cholesterol, LDL-cholesterol (Friedewald formula) and N-terminal prohormone of brain natriuretic peptide (NTproBNP) concentrations were measured on a Modular P800 analyser (Roche Diagnostics, Mannheim, Germany).

### CMR protocol

All participants underwent a cardiac MRI protocol using a clinical 3 Tesla Ingenia whole-body MR system (Philips Medical Systems, Best, the Netherlands) at baseline and follow-up. Subjects were scanned in supine position. The body coil was used for transmission, and reception was achieved with a 16-element anterior, and a 12-element posterior array. The heart was imaged in 2-chamber, 4-chamber and short-axis views with ECG-gated breath-hold balanced steady state free-precession cine imaging. Then, whole-heart 4D velocity encoded flow MRI was performed as described elsewhere [12]. For visualisation of prior myocardial scarring, a free-breathing high spatial resolution delayed enhancement phase-sensitive inversion recovery sequence was acquired after intravenous administration of gadolinium contrast material (0.3 mL/kg, Dotarem; Guerbet, Bloomington, USA) [13]. All images were blinded for study participant and occasion (baseline or follow-up). Image post-processing was performed using validated MASS software (LUMC, Leiden, the Netherlands). LV diastolic function comprised of early peak mitral annular septal tissue velocity (Ea in cm/s) which was analysed with the use of 4 chamber long-axis view. Early (E) and late (A) peak transmitral flow rate (in mL/s) and E/A ratio were analysed using 4D flow dataset with retrospective valve tracking [12]. E deceleration peak (Edec) was defined as the maximum downward slope of early peak flow rate. E (in cm/s, without background subtraction) divided by Ea is a validated estimate of LV filling pressure [14]. Short axis cine images were used to measure LV systolic function parameters: stroke volume, ejection fraction, cardiac output and cardiac index (cardiac output/body surface area). The LV systolic function parameter peak ejection rate was measured with 4D flow MRI. The heart rate during MRI scan was chosen to report because that heart rate most closely reflects cardiac dynamics as assessed with MRI. LV filling volume was analysed with 4D flow. LA volume was calculated using Simpsons rule [10] and then divided by body surface area to obtain LA volume index. LV end-diastolic volume (LVEDV), LV end-systolic volume (LVESV) and LV mass (LVM) were all obtained from short axis cine imaging studies. Parameters LV mass index (LV mass/body surface area) and LVMI/LVEDVI (LV mass index/LVEDV index) and LV compliance (LVEDV/E/Ea) were calculated.

### Study endpoints

Since integrative assessment of cardiac function encompasses both LV diastolic and systolic indices, all were marked as primary endpoint. However, for sample size calculation (see below) Edec and LV ejection fraction were used. Predefined secondary endpoint were blood pressure, body weight, HbA1c, LVEDV, LVESV, LVM, LVMI, LVMI/LVEDVI. Other pre-specified endpoints were creatinine and NTproBNP. Endpoints that were not predefined were heart rate, LV filling volume, LA volume, LA volume index and LV compliance. We chose to report these endpoints for interpretation purposes.

### Statistical analysis

Sample size was calculated based on a publication on the effects of pioglitazone on cardiac function parameters [15] (for Edec), and on a study describing the effect of GLP-1RA in patients with DM2 with heart failure with reduced ejection fraction [16] (for ejection fraction). We estimated that for a power of 90%, α of 0.05 and minimum expected difference of 25% (SD20%), we would need a sample size of 9 to 17 patients per treatment arm. Furthermore, taking into consideration that the study population in our trial would have a significantly better systolic function than the patients with heart failure studied in the trial mentioned above, differences might be smaller. Finally, assuming a 10% loss to follow-up, we aimed to include 25 patients per group. Data are shown as mean ± SD, or as median (interquartile range) when not normally distributed. For all presented study endpoints, we performed an intention-to-treat analysis of covariance (ANCOVA) of between-group differences of change from baseline with randomization arm as the independent variable and the baseline measurement of dependent variable as a covariate. Statistical analyses were performed using SPSS version 23.0 for Windows (IBM Corporation, Chicago, IL). A *P* value < 0.05 was considered statistically significant.

### Role of the funding source

Novo Nordisk (Denmark) funded this investigator-initiated study. Novo Nordisk had no role in the design of the study, data collection, data analysis, data interpretation, or writing of the report. All authors had access to all the data and final responsibility for the decision to submit for publication.

## Results

**Fig. 1:**
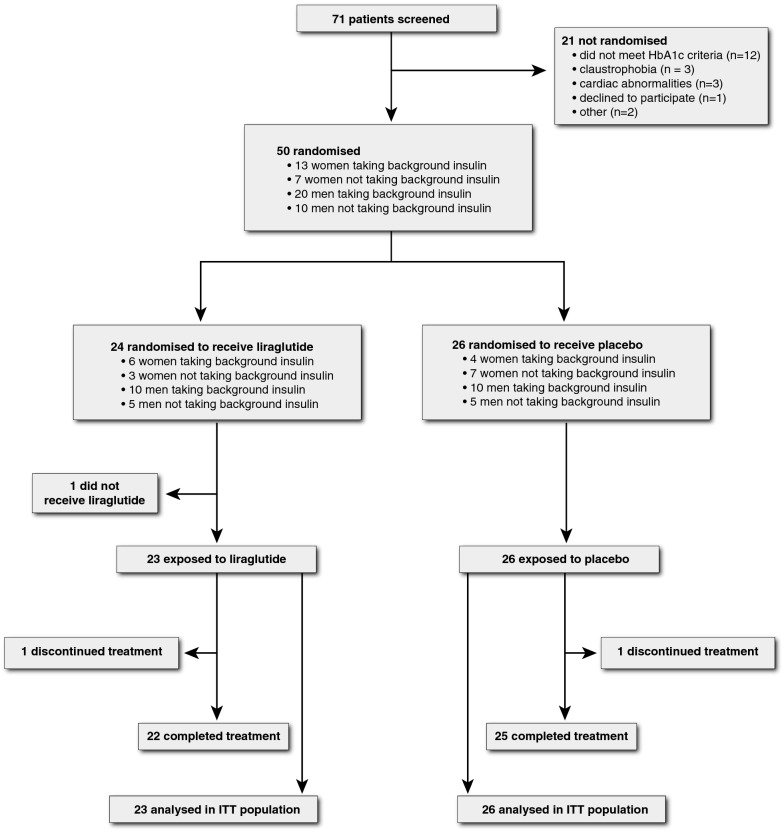
Trial profile. Patients were randomized with stratification according to sex and insulin use. One patient in liraglutide group withdrew consent before he ever received study drug. This patient was therefore not included in intention-to-treat analysis. In another patient assigned to liraglutide, withdrawal had taken place upon repeated hypoglycaemic events (on further examination this patient had positive anti-glutamic acid decarboxylase autoantibody titer and undetectable c-peptide levels consistent with type 1 diabetes mellitus). In the placebo group, one patient was lost to follow-up because he was in detention. All other patients reached end of study. *ITT* intention-to-treat

Participants were enrolled between December 2013 and September 2015 with last patient last visit in March 2016. Figure 1 shows the trial profile and baseline characteristics are shown in Table 1. Sex, insulin use, age, blood pressure, use of anti-hypertensive drugs, lipid levels, smoking history and glycaemic control were comparable in both groups. Liraglutide patients had slightly higher BMI (+ 1.0 kg/m^2^). There was an uneven distribution of nephropathy (9% in liraglutide versus 42% in placebo group). With regard to primary outcome measures there was slightly higher E/A ratio (+ 0.05) and lower E/Ea (− 0.6) in liraglutide versus placebo at baseline. In the liraglutide group uptitration was delayed in five patients versus none in placebo group, and study drug dose that patients used was 0.6 mg (n = 2); 1.2 mg (n = 3) and 1.8 mg (n = 18). In placebo group, no patient had delayed uptitration and all patients used 1.8 mg once daily. The cumulative prescribed study drug dose was 278.4 ± 45 mg in liraglutide versus 302.4 ± 13.8 mg in placebo with compliance of 98% (± 3) versus 96% (± 4).

**Table 1:** Baseline characteristics of trial population

	Liraglutide (n = 23)	Placebo (n = 26)
Demographics		
Age in years (SD)	60 (6)	59 (7)
Male	14 (61%)	15 (58%)
Diabetes duration in years (SD)	11 (6)	11 (7)
Diabetes complications		
Retinopathy, n	4 (17%)	2 (8%)
Nephropathy, n	2 (9%)	11 (42%)
Neuropathy	10 (44%)	7 (27%)
Macrovascular^a^	2 (9%)	0 (0%)
Clinical parameters		
Weight in kg (SD)	98 (14)	94 (13)
Body-mass index in kg/m^2^ (SD)	32.6 (4.4)	31.6 (3.4)
Systolic blood pressure in mmHg (SD)	141 (14)	141 (15)
Diastolic blood pressure in mmHg (SD)	86 (6)	87 (11)
Glycated haemoglobin A1c in % (SD)	8.4 (1.1)	8.2 (1.0)
Glycated haemoglobin A1c in mmol/mol (SD)	67 (12)	65 (10)
Serum creatinine in μmol/L (SD)	73 (19)	68 (17)
Urinary albumin/creatinine ratio in mmol/μg (SD)	1.0 (1.3)	5.0 (8.9)
Triglycerides in mmol/L (SD)	2.2 (1.5)	2.1 (1.1)
Total cholesterol in mmol/L (SD)	4.8 (1.0)	4.8 (1.0)
HDL-c in mmol/L (SD)	1.2 (0.2)	1.3 (0.4)
LDL-c in mmol/L (SD)	2.6 (0.9)	2.5 (0.9)
Smoking history		
Never smoked, n	10 (44%)	8 (31%)
Current smoker, n	4 (17%)	5 (19%)
Ex-smoker, n	9 (39%)	13 (50%)
Concomitant drug use		
Metformin dose in g/day (SD)	2.1 (0.7)	2.0 (0.5)
Sulfonylurea, n	6 (26%)	8 (31%)
Insulin, n	15 (65%)	17 (65%)
Anti-lipidaemic, n	21 (91%)	19 (73%)
Anti-hypertensive, n	18 (78%)	20 (77%)
LV diastolic function		
E in mL/s (SD)	331 (99)	325 (96)
A in mL/s (SD)	367 (79)	371 (70)
E/A ratio (SD)	0.95 (0.44)	0.90 (0.31)
Edec in mL/s^2^ × 10^−3^ (SD)	2.9 (0.9)	2.6 (1.2)
Ea in cm/s (SD)	6.0 (1.6)	6.0 (1.8)
E/Ea (SD)	7.3 (2.9)	7.9 (2.3)
LV systolic function		
Stroke volume in mL (SD)	81 (16)	76 (18)
Ejection fraction in % (SD)	55 (5.8)	55 (4.5)
Cardiac output in L/min (SD)	5.4 (0.9)	5.5 (1.0)
Cardiac index in L/min/m^2^ (SD)	2.5 (0.3)	2.6 (0.4)
Peak ejection rate in mL/s (SD)	442 (96)	415 (92)

*E* early transmitral peak flow rate, *A* late transmitral peak flow rate, *Edec* peak deceleration of transmitral early peak flow, *Ea* early peak mitral annular septal tissue velocity

^a^Macrovascular complications were cerebrovascular or peripheral artery disease and not cardiovascular

### Concomitant glucose-lowering drugs

In liraglutide group use of SUD decreased from 26% at baseline to 18% at 26 weeks, and the use of insulin decreased from 70 ± 46 to 54 ± 43 IU/day (percentage of participants on insulin therapy decreased from 65 to 64%). In placebo group use of SUD increased from 31 to 40%. Number of insulin users increased from 65 to 72% with average daily dose of 69 IU at baseline and 69 IU at 26 weeks.

### Anthropometric and laboratory values

Liraglutide group had significantly more weight loss than placebo group (− 4.3 ± 3.8 kg vs 0.1 ± 2.5 kg, p < 0.001). Systolic and diastolic blood pressure changes were not different amongst treatment groups (p = 0.63 and p = 0.23 respectively). In both liraglutide and placebo treated patients an improvement in glycaemic control was noticed. In liraglutide group HbA1c decreased 1.1 ± 1.0% (11.6 ± 11.1 mmol/mol) versus 0.7 ± 0.9% (7.7 ± 9.4 mmol/mol) decline in placebo group, with no significant difference between group changes (estimated mean treatment difference: − 2.9 with 95% CI from − 8.1 to 2.3 mmol/mol, p = 0.27). Serum creatinine slightly increased in both treatment groups but there was no difference between group changes (liraglutide: + 4 ± 5 μmol/L; placebo: + 4 ± 5 μmol/L, p = 0.69). NTproBNP levels declined from 45 ± 30 to 37 ± 18 pg/mL in liraglutide group, and increased in placebo group from 39 ± 29 to 45 ± 29 pg/mL, with estimated mean treatment difference of − 10 pg/mL with 95% CI between − 20 and 1 pg/mL, p = 0.07.

### Magnetic resonance imaging and spectroscopy

In one patient in the liraglutide group a small area of delayed contrast enhancement was noted in the inferoposterior basal segment. On further examination by cardiologist there was no sign of cardiac ischemia during exercise testing. All other patients had no late gadolinium enhancement.

**Fig. 2:**
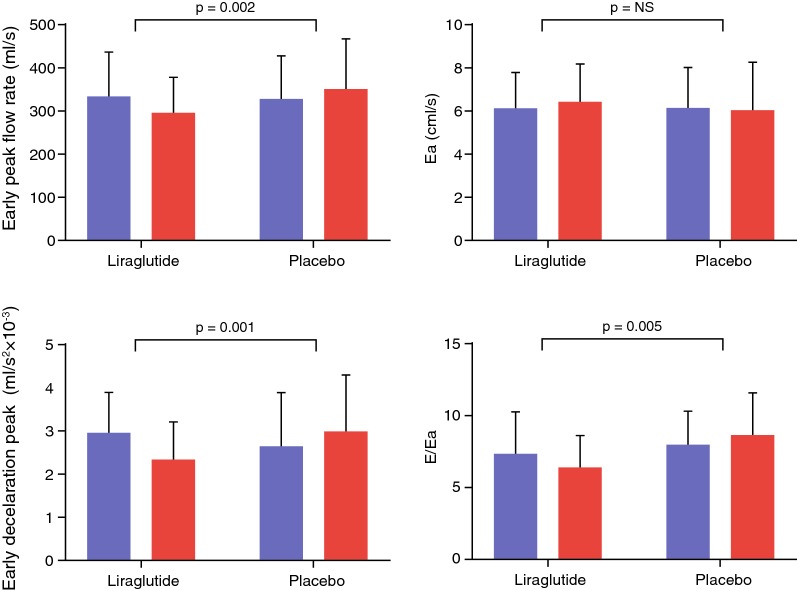
LV diastolic function. Bar graphs of MR-derived indices of LV diastolic function. Blue bars indicate baseline measurement and red bars follow-up. Ea reflects the early peak longitudinal annular motion that is dependent on LV myocardial relaxation. E/Ea is the MR estimate of LV filling pressure. *NS* not significant

**Fig. 3:**
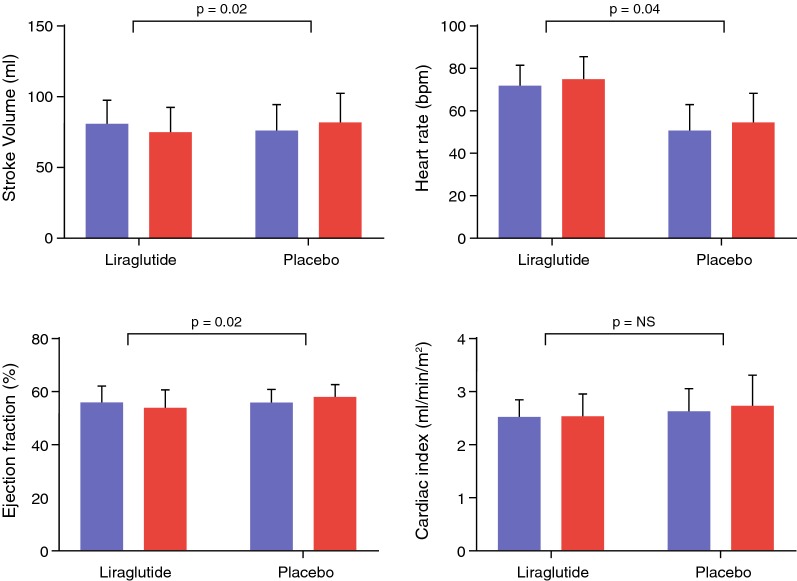
LV systolic function. Bar graphs of MRI-derived indices of systolic function. Blue bars indicate baseline measurement and red bars follow-up. In the liraglutide group stroke volume decreased, whereas cardiac index remained unchanged because of the increased heart rate. *Bpm* beats per minute

Primary endpoints are shown Table 2 and Fig. 2. LV diastolic function indices that changed significantly between groups were E, E/A ratio, Edec and E/Ea. All these parameters were reduced by liraglutide, as compared with placebo. A and Ea were not affected by treatment. LV systolic function parameters that changed significantly between groups were stroke volume and ejection fraction. Despite a reduction in these parameters, cardiac output and cardiac index did not change between groups, due to increased heart rate (Fig. 3).

**Table 2:** Primary outcome measures

	Mean (SD) change from baseline to 26 weeks		Mean (95% CI) changes from baseline (liraglutide vs placebo)	P value
	Liraglutide (n = 23)	Placebo (n = 26)		
LV diastolic function				
E in mL/s (SD)	− 33 (59)	23 (62)	− 56 (− 91 to − 21)	0.002
A in mL/s (SD)	31 (77)	23 (62)	3 (− 35 to 41)	0.88
E/A (SD)	− 0.19 (0.31)	− 0.00 (0.17)	− 0.17 (− 0.27 to − 0.06)	0.003
Edec in mL/s^2^ × 10^−3^ (SD)	− 0.6 (0.6)	0.3 (0.9)	− 0.9 (− 1.3 to − 0.4)	< 0.001
Ea in cm/s (SD)	0.4 (1.8)	− 0.2 (1.7)	0.4 (− 0.6 to 1.4)	0.40
E/Ea (SD)	− 0.9 (2.6)	0.6 (1.9)	− 1.8 (− 3.0 to − 0.6)	0.005
LV systolic function				
Stroke volume in mL (SD)	− 4 (13)	5 (12)	− 9 (−16 to −2)	0.02
Ejection fraction in (% (SD)	− 1 (5)	1 (5)	− 3 (−6 to − 0.1)	0.02
Cardiac output in L/min (SD)	0.0 (0.9)	0.3 (1.1)	− 0.4 (− 0.9 to 0.2)	0.21
Cardiac index in L/min/m^2^ (SD)	− 0.0 (0.4)	0.1 (0.5)	− 0.1 (− 0.4 to 0.1)	0.27
Peak ejection rate in mL/s (SD)	− 28 (89)	24 (82)	− 46 (−95 to 3)	0.07

Within group and between group changes in left ventricular diastolic and systolic function between baseline and 26 weeks (primary outcome)

*E* early transmitral peak flow rate, *A* late transmitral peak flow rate, *Edec* early deceleration peak of transmitral flow rate, *Ea* early peak mitral annular septal tissue velocity

**Fig. 4:**
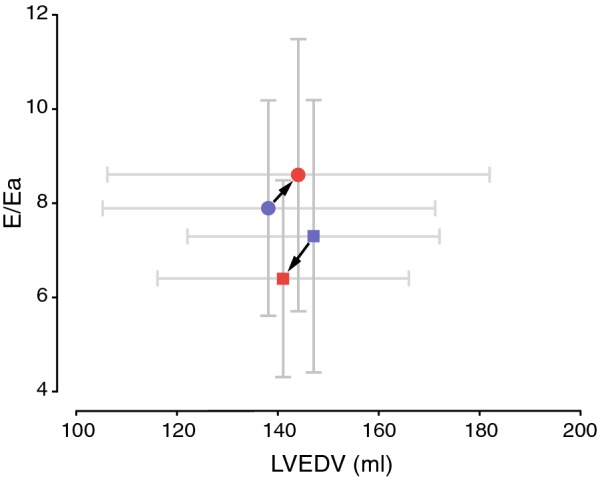
Pressure-volume relation. The LV filling pressure estimate E/Ea plotted against LV end-diastolic pressure (LVEDV). Liraglutide treatment (squares; blue = baseline, red = follow-up) results in a lower E/Ea and LVEDV, whereas placebo treated patients (circles; blue = baseline, red = follow-up) have higher E/Ea and LVEDV at follow-up then at baseline. Note that the shift in pressure volume curve is in opposite directions for liraglutide versus placebo. There was a tendency towards improved compliance in the liraglutide group

Table 3 displays non-primary outcome measures. In line with reduced stroke volume, the LV filling volume is also reduced in liraglutide as compared to placebo. Furthermore, LVM significantly decreased in liraglutide compared to placebo, but when corrected for reduced body surface area (LVMI) this difference did not persist. LVEDV was significantly reduced by liraglutide as compared to placebo treated patients. LV compliance showed a non-significant trend towards increased compliance in liraglutide versus placebo (Fig. 4).

**Table 3:** Heart rate and heart dimensions

	Liraglutide (n = 23)			Placebo (n = 26)			Mean (95% CI) changes from baseline (liraglutide vs placebo)	p value
	Baseline	26 week	Mean (SD) change from baseline	Baseline	26 week	Mean (SD) change from baseline		
Heart rate in bpm (SD)	72 (9)	75 (10)	4 (8)	77 (13)	76 (13)	− 1 (6)	4.3 (0.2 to 8)	0.04
LV filling volume in mL (SD)	82 (17)	76 (17)	− 5 (15)	74 (17)	82 (21)	7 (10)	− 11 (− 18 to − 3)	0.01
LA volume index in mL/m^2^ (SD)	36 (8)	35 (7)	− 1 (6)	32 (8)	34 (10)	1 (7)	− 2 (− 6 to 2)	0.38
LVEDV in mL (SD)	147 (25)	141 (25)	− 5 (14)	138 (33)	144 (38)	6 (16)	− 11 (− 20 to − 2)	0.02
LVESV in mL (SD)	67 (14)	66 (14)	− 0 (9)	62 (17)	63 (20)	1 (9)	− 1 (− 7 to 4)	0.69
LVM in g (SD)	107 (18)	105 (18)	− 2 (8)	108 (27)	110 (29)	4 (9)	− 6 (− 11 to − 1)	0.03
LVMI in g/m^2^ (SD)	49 (6)	49 (6)	− 0 (3)	50 (11)	52 (12)	2 (4)	− 1.5 (−3.6 to 0.6)	0.17
LVMI/LVEDVI g/mL/m^2^ (SD)	0.73 (0.10)	0.75 (0.11)	0.01 (0.07)	0.79 (0.14)	0.77 (0.14)	− 0.00 (0.08)	0.01 (− 0.03 to 0.06)	0.60
LV compliance (SD)	23.4 (10.4)	24.1 (8.3)	0.7 (9.7)	19.5 (8.0)	18.6 (8.2)	− 0.3 (6.8)	3 (− 1 to 7)	0.14

*bpm* beats per minute, *LA* left atrial, *LVEDV* left ventricular end-diastolic volume, *LVESV* left ventricular end-systolic volume, *LVM* left ventricular mass index, *LVMI* left ventricular mass index, *LVEDVI* left ventricular end diastolic volume index

### Safety

One patient in the liraglutide group developed edema after starting calcium channel blockers. There were no patients that developed (symptoms of) heart failure during the study. There were three serious adverse events that were not related to study drug use. Other adverse events were mild and predominantly of gastro-intestinal origin. There were no cases of acute pancreatitis during the study period.

## Discussion

This study shows that in patients with type 2 diabetes mellitus without prior cardiovascular disease, 6-month treatment with liraglutide improved E/Ea, as compared with placebo added to standard care. As such, liraglutide beneficially influenced a key pathogenic hallmark of HFpEF: left ventricular filling pressure. Liraglutide did not improve left ventricular myocardial relaxation (Ea). Liraglutide reduced left ventricular systolic function parameters stroke volume and ejection fraction, and these remained within normal range.

### Interpretation

Diabetes with or without the presence of hypertension is independently associated with abnormal LV diastolic filling pattern [1], i.e. diabetic cardiomyopathy. The asymptomatic stage can persist during years or decades, but once symptomatic heart failure has developed, progressive impairment of myocardial relaxation results in compensatory rise in E/Ea to ensure sufficient LV filling during diastole. These final stages of HFpEF are characterized by impaired quality of life and life expectancy [6]. The early asymptomatic stage with prevalence up to 50% [2] therefore seems a window of opportunity to reverse or delay progression of diabetic cardiomyopathy. However, there are no pharmacologic agents that have unequivocally shown benefit in HFpEF patients [17]. An anti-diabetic agent that positively affects HFpEF indices would therefore be of great clinical importance. In that regard, the observed reduction in E/Ea, is a promising prospect. Elevated filling pressure has been shown to independently predict progression of HFpEF in patients with DM2 [18]. Possible underlying cardiac pathologic mechanisms include wall stress, diffuse cardiac fibrosis and LV hypertrophy [19]. Liraglutide seems to positively affect these pathologic pathways, as evidenced by reduced E/Ea, LVM, and a trend towards improved LV compliance and NTproBNP levels, as compared to placebo. As such, it might be postulated that initiation of liraglutide treatment in the early asymptomatic stage of diabetic cardiomyopathy, could delay the onset of clinically significant HFpEF. With regard to systolic function, we hypothesize that reduced LV filling volume directly results in reduced stroke volume and ejection fraction. The modest decline of ejection fraction is not considered clinically relevant in this specific study cohort, because it remained within normal range [11]. Furthermore, cardiac output and cardiac index did not change due to rise in heart rate which is a well-documented finding in studies with GLP-1RA therapy [9].

### Possible mechanisms

The design of our study did not facilitate unravelling the mechanism by which liraglutide reduced E/Ea. There are several potential mechanisms to be addressed. First, liraglutide has been shown to have natriuretic [20] and vasodilatory [21] effect which could have lowered E/Ea by reducing cardiac preload. Second, the increase in heart rate could have affected LV diastolic filling pattern directly [22]. However, there are two reasons why we do not expect increased heart rate to be the causative effect of diastolic filling pattern changes: 1. the change in heart rate is relatively small in comparison to change in early filling; 2. a study in HFpEF patients using the selective sino-atrial node blocker ivabradine did not change E/Ea [23]. Lastly, a direct effect of GLP-1RA on the heart has been proposed as a mechanism to improve cardiac energy metabolism and thereby cardiac function. Although GLP-1 receptor is expressed in cardiomyocytes, to date it is uncertain what its exact function in humans is [24]. However, if liraglutide had improved myocardial relaxation, an increase in Ea would have been expected. Ea however did not change significantly, which suggests against a direct effect of liraglutide on cardiomyocyte relaxation properties. It is unlikely that weight loss explains the observed effect of liraglutide on LV diastolic function, because a previous study from our group [25] has shown that calorie restriction with significant weight loss increased E/A ratio probably as a result of improved LV relaxation and/or filling properties (since LV filling pressure remained unchanged). Another important cardiovascular effect of weight loss in the study by Hammer et al. was a significant decline in heart rate, which is a consistent finding in patients after weight loss. Therefore, the rise in heart rate in the present study is in keeping with the hypothesis that other mechanisms than weight loss are responsible for the observed changes in LV diastolic function.

### Comparison with literature

Some studies have investigated the effect of liraglutide on LV diastolic function. Nystrom et al. [26] found no change in echocardiography-derived indices of myocardial relaxation, E/Ea or LV ejection fraction in their non-blinded randomized study with 62 DM2 patients with subclinical heart failure receiving either liraglutide or glimepiride treatment. A double-blind randomized trial in 33 patients with DM2 who underwent a 16 week exercise program with addition of either liraglutide or placebo, showed significantly lower E/Ea in liraglutide treated patients [27]. Lastly, in two small non-randomized studies in patients with DM2, the effect of liraglutide was evaluated after 6 months using echocardiography. These studies showed a decrease in E/Ea [28] and improved Ea [28, 29]. The results of our placebo-controlled double-blind randomized study confirm the finding of some preliminary studies to date that 6-month therapy with liraglutide showing lower E/Ea. With regard to LV systolic function, not surprisingly, most studies have been performed in HFrEF patients with or without DM2 [30, 31]. In HFrEF GLP-1RA therapy has been shown to have no effect on LV systolic function, although there was a trend towards more frequent hospitalisation for heart failure in the study by Margulies et al. [31]. The small but significant decline in LV ejection fraction in our study is to our knowledge the first study reporting this effect of liraglutide in a DM2 population without prior cardiovascular disease.

### Clinical implications

The LEADER trial has shown that liraglutide reduces major adverse cardiovascular event rate (MACE) as compared to placebo in patients with DM2 [32]. The mechanisms responsible for GLP-1RAs beneficial effect on macrovascular diabetes complications remain to be established. Besides improvement of traditional cardiovascular risk factors, GLP-1RA treatment has been shown to reduce atherosclerotic plaque formation in mice by modulating macrophage phenotype [33], and reducing pro-inflammatory cytokines on a systemic level in conjunction with decreased leucocyte adhesion and extravasation into the vascular wall [34]. In addition, a direct effect of GLP-1RA on endothelial cells of injured mouse femoral arteries has been described that pointed towards suppression of restenosis via nitric oxide [35]. Although reduction in cardiovascular event rate is the most important factor for improving prognosis of patients with DM2, it is important to note that heart failure was not amongst the primary endpoints of the LEADER trial and other cardiovascular safety trials. As such, heart failure in patients with DM2 has been postulated to be the forgotten diabetes complication after microvascular and macrovascular complications [6]. This study shows that liraglutide has a significant effect on LV diastolic function. This study shows that short-term use of liraglutide is safe in DM2 patients with LV diastolic dysfunction without heart failure (symptoms). We argue against routine evaluation of cardiac function with imaging in these patients, because clinical implications for the individual patient are currently lacking. It is important to note that HFpEF patients with New York Heart Association class III or IV were excluded in the present study. Since these stages are accompanied by higher E/Ea, effects of GLP-1RA therapy in this group of patients cannot be extrapolated from our study. Since these patients are dependent on increased E/Ea for adequate LV filling, liraglutide might even risk exacerbation of heart failure symptoms and decompensation in this particular subgroup of patients.

## Limitations

First, the relatively low sample size was calculated to detect differences in Edec and LV ejection fraction. Other primary outcome measures were not included in sample size calculation. We did indicate the other indices of diastolic and systolic function as primary because they are very strongly causally linked to Edec and ejection fraction. Therefore, we did not correct for multiple testing. As a result of low sample size, there was an uneven distribution of BMI (slightly higher in liraglutide) and nephropathy (higher prevalence in placebo). Although BMI [36] and albuminuria [37] are associated with LV diastolic dysfunction, it is unlikely that this affects study outcome because differences are relatively small. Moreover, ANOVA analysis tests the differences between groups of within-group changes between baseline and follow-up, with correction for between-group differences at baseline. A second limitation is that we have chosen not to include LV diastolic dysfunction in the inclusion criteria of the study because there are no known cut-off values for LV diastolic dysfunction assessed with CMR. It is very likely that in our study population with mean diabetes duration of 11 years, poor glycaemic control, and high prevalence of hypertension, the vast majority of patients had LV diastolic dysfunction [1, 2, 6]. The third limitation regards the use of CMR. The reason CMR was used is that it is known for its excellent intra-observer reproducibility [11], and CMR is considered the gold standard for LV function and structure. CMR assessment of LV diastolic function has been shown to be a good alternative for echocardiography [10, 14]. It should be noted though that values for diastolic and systolic function as derived from CMR are not interchangeable with echocardiography [10, 11]. With regard to assessment of Ea, the relatively low temporal resolution of CMR as compared to echocardiography might have resulted in a lower power to detect significant differences. Another possible limitation is the relatively low sample size that does not facilitate reliable subgroup analyses.

## Conclusions

In conclusion, this study provides evidence that the GLP-1RA liraglutide influences both left ventricular diastolic and systolic function by unloading the left ventricle in patients with DM2. Because elevated left ventricular filling pressure is a driver for diabetic cardiomyopathy, an interesting hypothesis is that liraglutide could postpone the onset of HFpEF and concomitant morbidity and mortality. Liraglutide does not appear to have a direct effect on myocardial relaxation properties. The results of this study emphasize that larger studies specifically focusing on cardiac function are warranted in patients with DM2 with and without cardiovascular disease, including HFpEF. These studies will contribute to a more a complete understanding of cardiovascular benefit and safety of GLP-1RA therapy.

## Abbreviations

A: late transmitral peak flow rate
Bpm: beats per minute
CMR: cardiac magnetic resonance imaging
DM2: diabetes mellitus type 2
E: early transmitral peak flow rate
Ea: early peak mitral annular septal tissue velocity
Edec: peak deceleration of transmitral early peak flow
GLP-1RA: glucagon-like peptide-1 receptor agonist
HbA1c: glycated haemoglobin
HFpEF: heart failure with preserved ejection fraction
HFrEF: heart failure with reduced ejection fraction
ITT: intention-to-treat
LV: left ventricular
LVEDV: left ventricular end-diastolic volume
LVEDVI: left ventricular end-diastolic volume index
LVESV: left ventricular end-systolic volume
LVM: left ventricular mass
LVMI: left ventricular mass index
NTproBNP: N-terminal prohormone of brain natriuretic peptide
SUD: sulphonylurea derivative
