# Correction to: Effect of liraglutide on cardiac function in patients with type 2 diabetes mellitus: randomized placebo-controlled trial

**DOI:** 10.1186/s12933-019-0905-2

**Published:** 2019-08-09

**Authors:** Maurice B. Bizino, Ingrid M. Jazet, Jos J. M. Westenberg, Huub J. van Eyk, Elisabeth H. M. Paiman, Jan W. A. Smit, Hildebrandus J. Lamb

**Affiliations:** 1 Department of Radiology, Leiden University Medical Center, LUMC postzone C2S, Albinusdreef 2, 2333 ZA Leiden, The Netherlands; 2 Department of Medicine, Division of Endocrinology, Leiden University Medical Center, Leiden, the Netherlands; 3 Department of Medicine, Radboud University Medical Center, Nijmegen, The Netherlands

## Correction to: Bizino et al. Cardiovasc Diabetol (2019) 18:55 10.1186/s12933-019-0857-6

Following publication of the original article [1], the authors reported an error in Fig. 3. The bars in the upper right panel that represent heart rate in placebo treated patients is not correct. The revised Fig. 3 is given here.

**Fig. 3:**
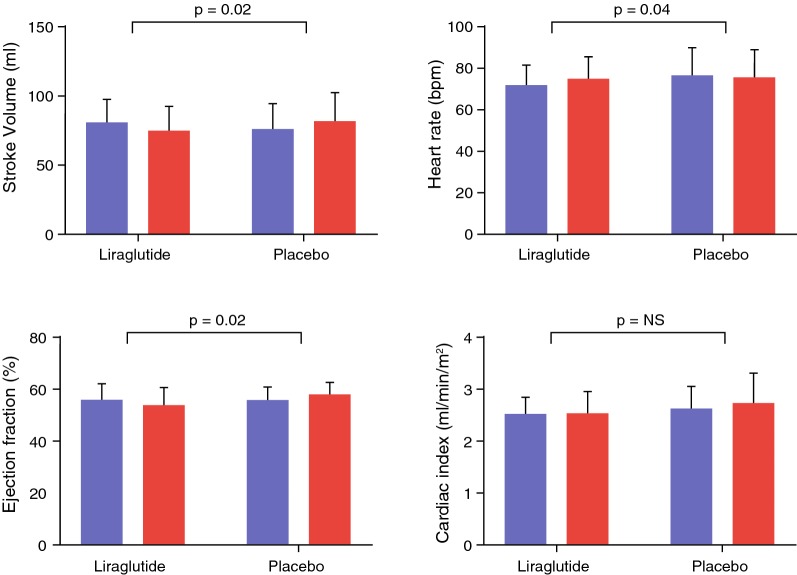
LV systolic function. Bar graphs of MRI-derived indices of systolic function. Blue bars indicate baseline measurement and red bars follow-up. In the liraglutide group stroke volume decreased, whereas cardiac index remained unchanged because of the increased heart rate. *Bpm* beats per minute

The authors wish to apologise for this error.
